# Nanoscale Undulation of Elastic Fields During Deformation Twinning in FCC Metals

**DOI:** 10.3390/ma19030585

**Published:** 2026-02-03

**Authors:** Di Qiu, Pengyang Zhao

**Affiliations:** 1 Materials Genome Institute, Shanghai University, Shanghai 200444, China; diqiu0319@shu.edu.cn; 2 Shanghai Frontier Science Center of Mechanoinformatics, Shanghai University, Shanghai 200444, China; 3 Department of Engineering Mechanics, School of Ocean and Civil Engineering, Shanghai Jiao Tong University, Shanghai 200240, China

**Keywords:** twinning, nickel, high-entropy alloys, nanostructure, phase-field model

## Abstract

**Highlights:**

**What are the main findings?**

- Geometrically nonlinear phase-field modeling is developed to investigate deformation twinning at finite strains.
- Critical shear stresses of ~7 GPa for Ni and ~4 GPa for CoCrFeMnNi high-entropy alloy are identified for triggering elastic undulations.
- Predicted elastic undulations exhibit a characteristic nanoscale wavelength of 1–2 nm.

**What are the implication of the main findings?**

- Stripelike undulations serve as a mechanistic precursor that governs the formation of finely twinned microstructures.

**Abstract:**

Finely twinned microstructures are widely observed in metals and alloys but the underlying formation mechanisms remain debatable. In particular, the role of internal stresses in promoting these inhomogeneous patterns is still not clear. By incorporating a geometrically nonlinear microelasticity theory into phase-field framework, we study the evolution of elastic fields resulting from the growing deformation twins (DT) at grain boundaries in fcc metals. Simulations in two model systems, i.e., Ni and CoCrFeMnNi (a high-entropy alloy), show that as the external applied stress increases, the internal elastic fields begin to develop undulations with stripelike patterns owing to the significant geometrical nonlinearity associated with DT. This elastic undulation, absent in linear modeling, is initially nonuniform inside the grain and becomes global and coarsened, exhibiting a characteristic wavelength of ~1–2 nm. The predicted elastic inhomogeneity leads to a stack of alternating crystal orientations favored by the undulating local stress fields. The resemblance of our predicted stress undulation and the stripelike patterns in experiments may suggest a universal mechanistic origin of the nanotwinned microstructures widely observed in deformation twinning and displacive transitions.

## 1. Introduction

Nanoscale inhomogeneities play an important role in promoting unprecedented structural and functional properties in materials [1,2,3]. A prototypical example is the fine mixture of regions with alternating crystalline symmetries/orientations (usually termed as twins), which are commonly observed during deformation twinning (DT) and martensite transformation (MT) in a wide range of metals and alloys [4,5,6,7]. The possibility of tailoring the characteristic features of the resulting nonuniform patterns via techniques such as electrodeposition, sputtering, and severe plastic deformation leads to a novel strengthening strategy, which has been successfully demonstrated in both conventional metals [2,3] and the recently developed high-entropy alloys [8]. Despite the fundamental and practical significance, mechanisms that control the emergence and evolution of these inhomogeneities (microstructures), appeared frequently as stacks of fine twins, remain unclear. From the energetic perspective, the stored energy density is usually assumed to be specified by the deformation gradients of different twins. Then, the postulation that the experimentally observed microstructures are “minimizers” of the resulting total energy gives rise to conditions that the system should satisfy in order to exhibit the desired microstructures [9,10]. However, models along this line are geometric and static in nature without accounting for the effect of internal stresses. These stresses, triggered by the external applied load and resulting from lattice incompatibilities, act as the driving force for displacive processes.

Regarding the kinetics, the formation of these finely twinned microstructures are usually attributed to the so-called “autocatalytic effect”, in which the already formed twin will promote the formation of new twins without the supply of additional driving forces [11,12]. While it is well-known that stress concentration occurs at the twin tips, it is also true that such an effect must be short-ranged as the twin plate can be viewed as a stack of dislocation loops whose stress fields decay as 1/$r^{3}$ (with r being the distance) [13]. Thus, it remains unclear how such short-ranged autocatalysis would control the long-range formation of finely twinned microstructures. Additionally, the proposed consecutive sequence of nucleation and growth in a highly cooperative manner lacks experimental evidence. Theoretical consideration so far is limited to the elastic stability of a stack of twins, which is presumed rather than predicted by the analysis [11,14]. Previous studies on DT in nanocrystalline metals are mainly focused on explanation of the atomistic twinning routes [15,16]; mesoscale understanding of the stress-driven, highly dissipative DT process in terms of its morphological characteristics, in particular, is currently missing.

More recently, Zhang et al. [17] have demonstrated experimentally that DT in Al alloys can be activated by tuning generalized planar fault energies and suggested that the accommodation of localized elastic strain at grain boundaries, which arises during the growth of deformation twins, is critically mediated by grain rotation processes. In particular, their observation is characterized by uniplanar, parallel nanoscale DT, with a thickness of ~1–3 nm, occurring specifically in grains sized between 20 and 40 nm. In fcc high-entropy alloys like CoCrFeMnNi, experimental studies [18] have also demonstrated that intensive severe plasticity-induced DT significantly contributes to microstructure refinement, but the exact role of DT and its underlying formation mechanisms remain unclear. In fact, nanoscale displacive transformation (e.g., martensite or DT) is one of the important microstructural features that have been utilized to improve the mechanical properties of high-entropy alloys [19]. Qiang et al. [20] recently employed molecular dynamics simulations to reveal that inhomogeneous internal stress/strain fields induced by a dual-gradient (grain size–twin thickness) architecture in CoCrFeMnNi can regulate dislocation–DT interactions to achieve uniform deformation and synergistic strengthening–toughening. To advance the understanding of nanoscale DT from an internal stress perspective, it is essential to investigate its formation kinetics at the mesoscale, bridging atomic-scale mechanisms and macroscopic mechanical behavior. In this work, pure Ni and CoCrFeMnNi are utilized as representative FCC systems to evaluate how variations in elastic properties and lattice parameters influence the DT evolution.

In this study, we use phase-field (PF) modeling to investigate the physical origin of the finely twinned microstructures in DT. Simulation results suggest that during the growth of nanoscale DT, the elastic fields in the matrix can develop undulations with stripelike patterns. These undulations divide the material globally into alternating stripes favoring different crystal orientations and may be responsible for the formation of widely observed nanotwinned microstructures.

## 2. Materials and Methods

A recently developed PF model [21] is used to simulate the growth of DT in a bicrystal. Given a deformation x= χ(X, t) of a displacive process, the model assumes a multiplicative decomposition of the deformation gradient, $F\equiv \partial \chi (X)/\partial X=F_{e}F_{i}$, where $F_{i}$ and $F_{e}$ represent the inelastic and elastic deformations, respectively, and transform a material line element dX successively to an intermediate configuration element *d***x**^−^ and the current configuration element dx. An order parameter (OP) field η(X) is employed, which takes the value of 0 inside the matrix, 1 inside the twin, and between 0 and 1 at the interface. Since the current study is focused on the early stage of deformation twinning at a grain boundary, only a single twin variant is considered in each grain. It is noteworthy, however, that our phase-field model, based on the rigorously derived multivariant formulation in Ref. [21], can be readily extended to simulate twin–twin interactions. This capability is particularly relevant for nanomaterials, where twins in nanograins are likely to encounter one another earlier and more frequently than in conventional coarse-grained materials.

A *kinematic* description of the DT microstructure is achieved by

(1) $$F_{i}=\left(1-\phi \left(\eta \right)\right)I+\phi \left(\eta \right)F_{i}^{0}$$

where $F_{i}^{0}$ is the homogeneous deformation gradient associated with DT and $\phi (\eta )=3\eta^{2}-2\eta^{3}$ is an interpolation function [22]. In face-centered cubic (fcc) crystals, by choosing a Cartesian basis ${\left\{e_{i}\right\}}_{i=1}^{3}$ with $e_{2}||[\bar{2}11](\eta_{1})$ and $e_{3}||[111](K_{1})$, we have $F_{i}^{0}=I+\gamma_{0}e_{2}⊗e_{3},$ where $\gamma_{0}=1/\sqrt{2}$ [23]. Note that the current model ignores the effect of discrete, microscopic dislocation activities, which can be viewed as a secondary relaxation mechanism relative to the primary microelasticity resulting from $F_{i}^{0}$. This plastic relaxation effect attributed to dislocations can be effectively, albeit implicitly, accounted for within our model by adjusting or relaxing the eigen-deformation (twinning shear) associated with the DT.

The total Helmholtz free energy is formulated as

(2) $$\Psi =\int_{V}A_{1}\eta^{2}{\left(1-\eta \right)}^{2}dV+\kappa :\left(\nabla \eta ⨂\nabla \eta \right)dV+\frac{1}{2}\int_{V}\left(F_{i}^{-T}E_{e}F_{i}^{-1}\right):C:\left(F_{i}^{-T}E_{e}F_{i}^{-1}\right)dV$$

where the integral and field operators all correspond to the initial (reference) configuration. The first integral contains the chemical and interfacial contributions, where $A_{1}=12\Gamma /l$ and κ under ${\left\{e_{i}\right\}}_{i=1}^{3}$ is a diagonal tensor with $\kappa_{11}=\kappa_{22}=4\kappa_{33}=3\Gamma l/4$. Here, Γ and l are the energy and thickness of the coherent twin boundary (CTB) [22]. The second integral represents elastic strain energy at finite strains [21], where $E_{e}=(F^{T}F-F_{i}^{T}F_{i})/2$ and C is the elastic stiffness tensor. The grain boundary is currently treated as a sharp dividing surface that creates elastic heterogeneity. The complexity of grain texture, arising from the initially inhomogeneous Ω and grain reorientation accompanying DT, is addressed by linearly interpolating materials parameters (e.g., elastic stiffness tensor) used in Equation (2) in the same manner as $F_{i}$ in Equation (1). The microstructure evolution is modeled using the time-dependent Ginzburg–Landau kinetic equation dη(X)/dt=−M(δΨ/δη), where M is a mobility constant. In calculating the functional derivative, the first term in Equation (2) is straightforward, and for the second, the result has been shown to be $-F_{i}^{-T}F^{T}FS:(dF_{i}/d\eta )$, where S is a stress tensor determined by C and deformation gradients (see [21]).

Two fcc metals exhibiting nanoscale DT, i.e., pure Ni and equimolar CoCrFeMnNi are chosen, with the relevant properties listed in Table 1. The direct measurement of CTB thickness l is currently not available but essentially controls the relative ratio between the interfacial and elastic energies; together with the grid length $l_{0}$, it defines the reduced gradient coefficient $\kappa_{11}^{*}=(l/4l_{0})$ [21]. According to the atomic-resolution transmission electron microscopy [6], as well as atomistic simulations [24] of DT in FCC alloys, l can only be a fraction of the interplanar spacing $d_{\left\{111\right\}}$ due to the coherent nature of the boundary. We take $l=2l_{0}=0.02\;nm$ (~$0.1d_{\left\{111\right\}}$) in the following 2D simulations and $l=l_{0}=0.1\;nm$ (~0.5$d_{\left\{111\right\}}$) in the 3D ones. (Note that so long as the appropriate deformation gradients are used, other systems such as bcc and hcp metals can all be described using this framework [21].)

The growth of a pair of stable twin nuclei, initially placed at a bicrystal interface (a 10° tilt boundary about $e_{1}$), is first simulated using a 2048 × 2048 (20.48 × 20.48 nm) computational grid in the $e_{2}-e_{3}$ plane, with periodic boundary condition prescribed along all $e_{i}(i=1\ldots 3)$. (This computational grid is sufficiently large for the current purpose as the experimentally observed DTs in Ni and HEAs all exhibit an inter-twin spacing about a few times the twin thickness of ~10 nm [7,28].) Under a constant applied shear stress $\tau_{a}$ along $\eta_{1}$ in $K_{1}$ plane, the twin nuclei can grow into the grain interior, resulting in continuous evolution of elastic fields.

## 3. Results

### 3.1. Elastic Stripes and Boundary Morphology

**Figure 1:**
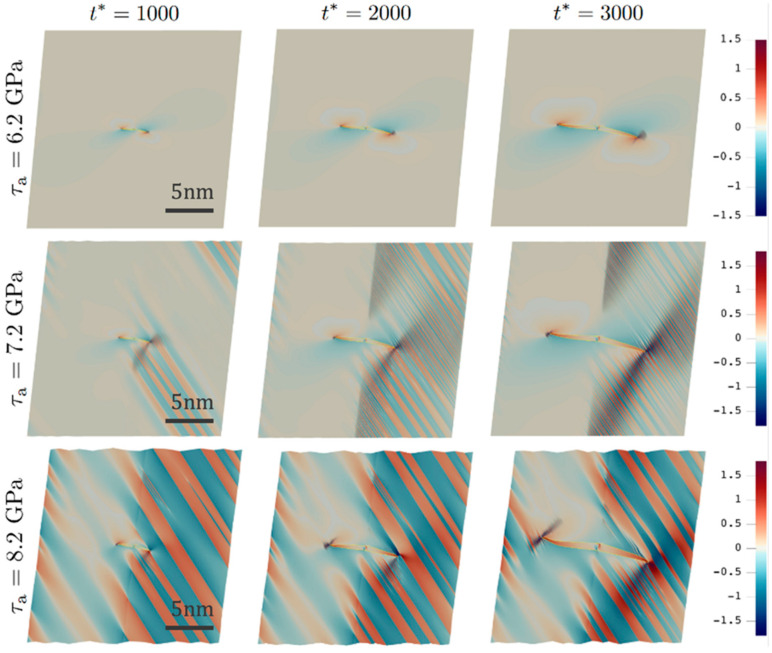
Snapshots of the deformed configuration colored by Cauchy stress $\sigma_{22}$ during DT (yellow lines) growth in Ni at different simulation time $t^{*}$ (row-wise) under various applied shear stresses $\tau_{a}$ (column-wise); (colorbar unit: $A_{1}$).

Figure 1 shows the representative evolution of the elastic fields by plotting the snapshots of the Cauchy stress component $\sigma_{22}$ at different times in various simulations. It is shown that stripelike patterns are developed globally for higher applied shear stresses of 7.2 and 8.2 GPa, whereas for the case of 6.2 GPa, the stress field exhibit the typical concentration at the twin tips and a smooth variation in the matrix. At $\tau_{a}=7.2$ GPa, the stripelike undulation consists of both short-range and long-range aspects, indicated by the patterns with different stripe widths shown in the middle column of Figure 1.

In fact, a short-range undulation begins to develop at the twin tip in one grain at $t^{*}=3000$ for $\tau_{a}=6.2$ GPa, but is highly localized as compared with that for $\tau_{a}=7.2$ GPa. At $\tau_{a}=8.2$ GPa, the undulation becomes significantly coarsened with much larger stripe widths. In addition, the initially flat boundaries normal to the vertical direction ($e_{3}$) adopt a zigzag morphology in line with the undulation, suggesting the highly dissimilar elastic deformation states in two adjacent stripes. A closer look at the results of $\tau_{a}=7.2$ GPa also finds this zigzag morphology, but at a smaller length scale due to the much finer undulations developed there; it is initially localized in regions at which the stripe pattern terminates and starts to spread out as the undulation continues to develop globally. Note that the simulated twin boundary at $\tau_{a}=8.2$ GPa becomes wavy near the tip region, which has also been observed experimentally in DT and MT [29,30,31].

### 3.2. Three-Dimensional Simulations and Fourier Analysis

**Figure 2:**
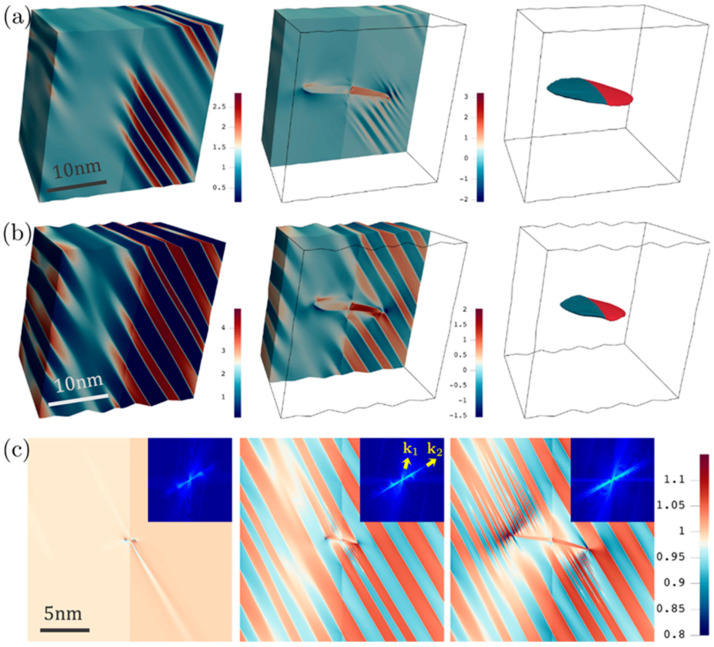
(**a**,**b**) Snapshots of the deformed configuration during DT in CoCrFeMnNi for $\tau_{a}$ = 5 GPa and 6 GPa, respectively; the surface representation is colored by Cauchy stress $\sigma_{32}$ (in units of $A_{1}$) and the twin morphology is colored by the second Euler angle. (**c**) Reference configurations at different DT stages colored by det(F), with the inset showing the corresponding Fourier transformed images.

The development of elastic undulations is also confirmed by 3D simulations of DT in CoCrFeMnNi using a 256 × 256 × 256 (25.6 × 25.6 × 25.6 nm) computational grids, which are shown in Figure 2a,b for $\tau_{a}$ = 5 and 6 GPa, respectively. The stress-dependence and nonuniform aspects of the elastic modulation, resulting zigzag boundary, and wavy CTB are all present. These stripelike patterns are also found to be oriented roughly along the same direction as that in the 2D simulations of Figure 1. To determine this direction, det(F) is plotted in Figure 2c using the results from 2D simulations for CoCrFeMnNi at $\tau_{a}$ = 4.6 GPa, together with the corresponding Fourier transformed images (insets in Figure 2c). In particular, the quantitative characterization of the elastic undulations was performed using a Fast Fourier Transform (FFT) on the scalar field of det(F). Two primary wave vectors were identified in the power spectrum $k_{1}$, which represents the geometric orientation of the primary twin, and $k_{2}$, which represents the periodic elastic undulations. The dominance of the undulation patterns is reflected in the significantly higher intensity observed along $k_{2}$ compared to $k_{1}$. Temporal analysis shows that as the twin grows, intensities emerge at larger wave vectors along $k_{2}$, capturing the transition from coarse structures to the fine-scale undulations observed at higher applied stresses.

It is also shown that $k_{1}$ is deviated from the K1 direction by ~10°, which is due to the elastic distortion at the twin tip [21], and $k_{2}$ makes an angle of ~40° from $k_{1}$. The much higher intensities along $k_{2}$ reflect the clear predominance of the undulation patterns.

In addition, high intensities start to appear at larger wave vectors along $k_{2}$ as the twin grows, suggesting the presence of fine-scale features, which correspond to the development of fine undulations emanating from the twin tips in both grains. Because of the 10° misorientation, the newly formed fine undulation in one grain is not exactly parallel to the already formed coarse undulation, resulting in a new high-intensity direction slightly deviated from $k_{2}$. These properties of elastic undulations are also present in the results of Ni (Figure 1). Note that in the presence of a GB, additional local stress contribution due to heterogeneous elasticity will be produced. It is shown in Figure 2 (as well as in Figure 1) that the onset and degree of undulation are different in the two grains, suggesting the effect of grain texture on DT behavior in different grain interiors. It is also emphasized that the current undulation cannot be captured by a linearized small-strain phase-field model, which fails to produce the deviation of twin boundary at the tip [21] and hence cannot obtain the local waviness that is responsible for the onset of the reported undulation.

### 3.3. Quantitative Characterization of Microstructure

**Figure 3:**
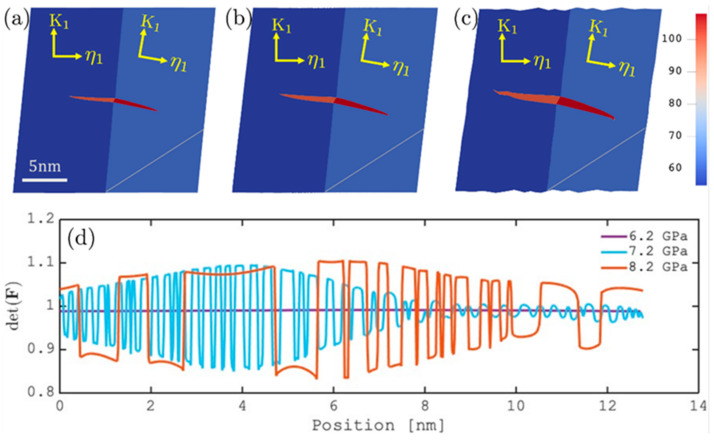
(**a**–**c**) Deformed configurations at $t^{*}=3500$, respectively, $\tau_{a}$ = 6.2, 7.2, and 8.2 GPa, colored by the second Euler angle (Bunge convention in units of °). (**d**) Variation in det(F) along the probe (white) lines shown in (**a**–**c**).

To obtain some quantitative understanding, we take the deformed configurations at a later stage of the simulations (corresponding to Figure 1), where the DT morphology is characterized by the crystal reorientation in Figure 3a–c. A probe line (roughly along $k_{2}$) inside the matrix is then chosen to inspect the variation in elastic fields. Figure 3d plots the resulting det(F) vs. position, which is characteristic of all elastic tensor components since the matrix undergoes pure elastic deformation.

**Figure 4:**
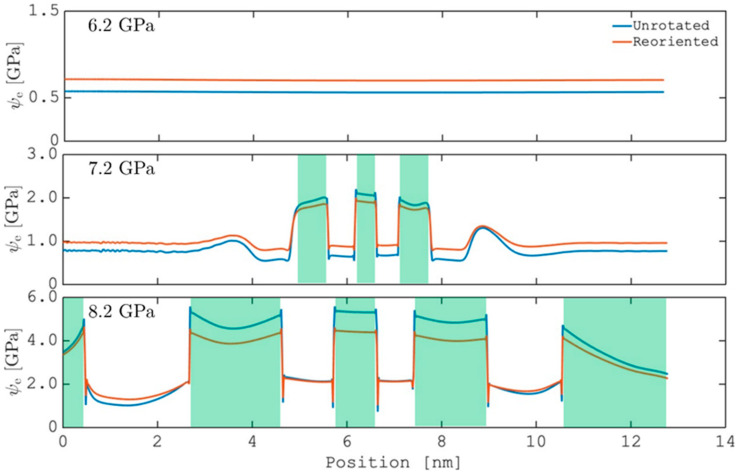
Elastic energy density $\Psi_{e}$ along the probe lines in Figure 3, calculated using the results at $t^{*}=1000$ in Figure 1 and considering both the unrotated and reoriented orientations. The regions corresponding to the twin orientation are colored in green.

It is then estimated that the undulation exhibits characteristic stripe widths about 1–2 nm (5–10d{111}) at $\tau_{a}=8.2$ GPa and 0.1–0.2 nm (about one $d_{\left\{111\right\}}$) at $\tau_{a}=7.2$ GPa, which are also consistent with the results of 3D simulations (Figure 2a,b). Since the DT nucleation in fcc metals generally involves only a few atomic layers [23,24,32], undulations at these characteristic lengths are expected to be of great significance to the subsequent dynamics of DT. In addition, undulations in the elastic fields should play a dominant role in controlling the evolution of DT morphology, as the models of nucleation, growth, and twin–twin interaction all involve the local stress as a major driving force [23,33]. As a quantitative demonstration, Figure 4 plots the local elastic energy density $\Psi_{e}$ along the probe lines in Figure 3, which is then reduced to $\Psi_{e}=\frac{1}{2}E_{e}:C:E_{e}$ with $E_{e}=(F^{T}F-I)/2$. Since DT is essentially a way of relaxing the local stress via reorienting the crystal, both the unrotated (matrix) and reoriented (twin) crystal orientations are considered when calculating $\Psi_{e}$ in Figure 3. It is shown that, in the absence of elastic undulations, $\Psi_{e}$ always assumes a lower value for the matrix orientation and DT is thus not favored by the corresponding stress fields. As elastic undulations start to be developed in part of the matrix, the twin orientation can lead to a lower $\Psi_{e}$ inside certain stripes, indicated as the colored intervals in Figure 4. These elastic stripes, where DT is expected to be promoted subsequently, are found to form an alternating sequence with stripes favoring the matrix orientation. This stack of alternating crystal orientations favored elastically is maintained as the undulation becomes global and coarsened with increasing the applied stress.

## 4. Discussion

The current simulation results suggest that DT in fcc metals can result in undulating elastic fields with stripelike patterns. It is found that the emergence of these elastic undulations depends highly on the magnitude of the external applied stress. At sufficiently large applied stresses, the elastic undulations extend globally right at the early stage of DT when the twins are only a few nanometers in length and exhibit a characteristic stripe width of 1–2 nm. Interestingly, in a recent experimental study of CoCrFeMnNi, uniplanar, parallel nanoscale DT, with a thickness of ~1–3 nm, are frequently observed particularly in nanoscale grains [18]. As the applied stress is decreased below a critical level, the elastic undulations cannot be developed throughout the growth of DT. This critical stress depends on the material considered and is found through systematic simulations to be ~7 GPa for Ni and ~4 GPa for CoCrFeMnNi. Based on the previous analysis in Figure 4, this critical stress may correspond (or relate) to the onset of forming a large population of secondary DTs in the grain scale. In many metals (including Ni and CoCrFeMnNi), it has been commonly observed that secondary DTs usually form a stack with extremely fine (nanoscale) thickness and spacing [7,34], which is consistent with the morphology of the simulated elastic undulations.

In addition, secondary DT only occurs in experiments when sufficient plastic deformation has been accumulated but with a little increase in the flow stress, which is also not well-understood. According to the current work, the role of plastic deformation is to create enough stress concentrations with sufficient strengths (roughly measured as the ratio between local and far-field stresses) in order to meet the critical stress for developing elastic undulations, which, according to our simulations, is much higher than that for forming a stable twin nucleus [7]. Also note that secondary DT evolves nonuniformly over different grains in experiments, which is reflected in our simulations as the emergence of elastic undulations in one grain but not in the other (Figure 1 and Figure 2).

The stripelike patterns of the simulated elastic fields represent the so-called cylindrical undulation, with the cylindrical axis being approximately normal to $k_{2}$ in Figure 2c. It has been determined previously that $k_{2}$ is inclined by ~40° with the K1 of the primary twin. Note that there are two DT variants for a given $\langle 110\rangle$ zone, which, in our case, are related by a rotation of ~70° around $e_{1}$. Thus, there seems to be a ~30° deviation between the cylindrical undulation and the secondary twin variant. However, due to the significant twinning shear of ~0.7, there is a rotation of ∼20° involved in the simple shear $F^{0}$, which may be accommodated by the elastic distortion of the surrounding matrix [35] and hence compensate for a major part of the deviation. (Note that this rotation of ~20° corresponds to the polar decomposition of $F^{0}$ and should be distinguished from the misorientation angle between the DT and matrix.) This consideration, together with the analysis on Figure 4, suggests that these cylindrical elastic undulations may effectively activate the secondary DT system.

The applied stresses used in our simulations, namely 6~8 GPa for Ni and 5~6 GPa for CoCrFeMnNi, appear to be very high. Nevertheless, it has been found experimentally that DT takes place at stresses order-of-magnitude higher than slip deformation in FCC alloys and increases significantly by reducing the grain size [33,36]. For nanocrystalline nickel, it has been shown experimentally that after sufficient strain hardening, a stress as high as 2~3 GPa is required to activate further slip deformation [5] and DT has been observed to be a competitive plastic mode upon strain-hardened significantly [30]. Considering that, in reality, local stress concentrators (e.g., dislocations and microcracks) can easily amplify the stress up to about three times of the average stress, the applied stress used in our simulation is actually quite reasonable. Similar scenarios exist in molecular dynamics simulations of DT, where stresses close to the theoretical critical shear strength are required to activate DT [37].

Regarding the nature of the reported undulation, the fact that stripelike elastic fields always initiate at the wavy tip of DT suggests that lattice rotation due to passage of twinning dislocations should be the underlying cause. This lattice rotation is incorporated in the current model via the usage of geometrically nonlinear finite-strain theory. Indeed, if linearized infinitesimal strain theory was employed to formulate the elastic energy, the resulting DT configuration will never exhibit waviness, i.e., deviation of the coherent twin boundary from the K1 plane [21].

The practical implication of the reported elastic undulations lies in the design and optimization of nanostructured materials. By providing the critical stress thresholds for the onset of global elastic undulation, our model offers a predictive tool for controlling the density and distribution of nanotwins during manufacturing processes such as cryo-forging, surface mechanical attrition treatment (SMAT), or high-pressure torsion [18,19,38]. It is expected that one may utilize these stress-based criteria to tune processing parameters (e.g., strain rate and temperature) to ensure that local stress concentrations effectively trigger the “elastic precursor” needed for extensive twinning-induced refinement.

Finally, we note that the predicted elastic undulations are a consequence of the nonlinear elastic deformation subjected to the combined effects of the external applied load and internal crystal reorientation due to DT, which are also expected to be of some significance to shear-dominant MT. It is emphasized that unlike the inhomogeneous states of OPs that have been reported in many PF simulations of DT and MT, the undulating elastic fields within the pre-transformation region are rarely reported and may be closely related to long-standing phenomena of pre-martensitic transformation, which according to the recent experiments [39] represents an inhomogeneous lattice strain state with a cylindrical undulation at nanoscale. It is also worth noting that, owing to the continuum nature of phase-field method and elasticity theory, the predicted fine undulation (with a stripe width of 0.1–0.2 nm) in Figure 3d is better to be understood as an indicator for transition to more profound (coarser) undulations at higher applied stresses, as shown in Figure 3d. Since dislocations have not been considered, the effect of elastic driving force might be overestimated in the current simulations. The model can be further refined to include the role of dislocations in our future study.

## 5. Conclusions

This work carried out phase-field modeling of deformation twinning at finite strains in two fcc metals. The major findings and the new scientific knowledge obtained are summarized as follows:

(1) We identified that the growth of nanoscale DT leads to the development of undulating elastic fields with stripelike patterns. This predicted inhomogeneity creates a stack of alternating crystal orientations favored by the local stress fields, suggesting a universal mechanistic origin for the nanotwinned microstructures widely observed in experiments.
(2) Systematic simulations determined that the emergence of these global elastic undulations depends on the magnitude of the applied stress. The critical shear stress required to trigger this phenomenon is ~7 GPa for pure Ni and ~4 GPa for the CoCrFeMnNi high-entropy alloy.
(3) At sufficiently high stresses, the elastic undulations exhibit a characteristic stripe width (wavelength) of 1~2 nm. This nanoscale dimension aligns with experimental observations of extremely fine secondary nanotwins and serves as an elastic precursor for subsequent plastic pattern development.
(4) It is demonstrated that these undulations are a direct consequence of nonlinear elastic deformation subjected to combined external loads and internal crystal reorientation. It is shown that the linearized small-strain models fail to capture the DT boundary waviness at the tip, and therefore cannot predict the onset of these reported elastic undulations.

## Figures and Tables

**Table 1:** Elastic constants ($c_{11}$, $c_{12}$, $c_{44}$, in GPa) and the CTB energy ($\gamma_{t}$, in mJ/m^2^) (^a^ [25]; ^b^ [26]; ^c^ [27]).

Material	$c_{11}$	$c_{12}$	$c_{44}$	$\gamma_{t}$
Ni	246.5 ^a^	147.3 ^a^	124.7 ^a^	43 ^a^
CoCrFeMnNi	172.1 ^b^	107.5 ^b^	92 ^b^	16 ^c^

## Data Availability

The data presented in this study are available on request from the corresponding author. The datasets generated and analyzed during the current study are not immediately publicly available because they are integral to an ongoing research program. Premature release could compromise the integrity of our planned analyses and the publication rights of the research team members involved in these continuing projects.
